# Peculiarity of American Eyes

**Published:** 1888-11

**Authors:** 


### SELECTIONS.

### Peculiarity of American Eyes.

The efforts of the War Department to secure a field glass of greater power than the one they now use, has disclosed the fact that the eyes of the average American are closer together than those of men in foreign countries. The double glass, known as the field glass, now used, is weaker than that used in the armies of Europe. The only glass they can get of sufficient power is a single spy-glass, which is defective, in that it does not take in a broad enough field. This is a very serious defect in the equipment of the American army, but there seems to be no immediate prospect of its correction, because our eyes are too close together. Some of the colored troops may be able to use a different glass, but the white soldier can not overcome this national peculiarity.

